# GTXOP: A Game Theoretic Approach for QoS Provisioning Using Transmission Opportunity Tuning

**DOI:** 10.1371/journal.pone.0062925

**Published:** 2013-05-01

**Authors:** Mahdieh Ghazvini, Naser Movahedinia, Kamal Jamshidi

**Affiliations:** Department of Computer Engineering, Faculty of Engineering, University of Isfahan, Isfahan, Iran; Hungarian Academy of Sciences, Hungary

## Abstract

In unsupervised contention-based networks such as EDCA mode of IEEE 802.11(e)(s), upon winning the channel, each node gets a transmission opportunity (TXOP) in which the node can transmit multiple frames consequently without releasing the channel. Adjusting TXOP can lead to better bandwidth utilization and QoS provisioning. To improve WLAN throughput performance, EDCA packet bursting can be used in 802.11e, meaning that once a station has gained an EDCA-TXOP, it can be allowed to transmit more than one frame without re-contending for the channel. Following the access to the channel, the station can send multiple frames as long as the total access time does not exceed the TXOP Limit. This mechanism can reduce the network overhead and increase the channel utilization instead. However, packet bursting may cause unfairness in addition to increasing jitter, delay and loss. To the best of the authors’ knowledge, although TXOP tuning has been investigated through different methods, it has not been considered within a game theory framework. In this study, based on the analytical models of EDCA, a game theoretic approach called GTXOP is proposed to determine TXOP dynamically (i.e. according to the dynamisms of WLAN networks and the number of nodes in the network). Using GTXOP, each node can choose its TXOP autonomously, such that in addition to QoS improvement, the overall network performance is also improved.

## Introduction

Due to simple deployment and low cost, the IEEE 802.11 is considered as the de facto standard for Wireless Local Area Network (WLANs) technology. The IEEE 802.11 Medium Access Control (MAC) uses two main access mechanisms to access the wireless media: The first is Point Coordination Function (PCF) which is a contention free medium access mechanism and the other, Distributed Coordination Function (DCF) is a contention-based approach. Simple implementation of DCF renders this function as the most common access control mechanism for wireless networks. However, DCF does not provide any service differentiation and Quality of Service (QoS) operation to guarantee throughput or delay. As a result, in this mechanism, flows may experience uncontrolled delays under high load conditions [1]. The IEEE 802.11 working group introduced the IEEE 802.11e standard to differentiate the traffic flows in the network in order to improve the IEEE 802.11 standard to support QoS in WLANs. The IEEE 802.11e standard specifies a new medium access method called Hybrid Coordination Function (HCF) which consists of a contention free channel access method, *HCF Controlled Channel Access* (HCCA), and a contention-based channel access method called *Enhanced Distributed Channel Access* (EDCA) [2].

Several Access Categories (ACs) per each node are defined in the IEEE 802.11(e) (s) EDCA protocol. Each AC has its own queue and channel access differentiation parameters such as the Arbitration Inter-frame Space (AIFS), the Contention Window (CW) and the Transmission Opportunity (TXOP).

AIFS tuning operates by shortening or expanding the period in which a wireless station has to wait before it attempts to access the channel. A shorter AIFS period means that a frame can be transmitted with low latency, which is important for delay sensitive traffics. In case of a busy channel, or a transmission collision, a back off process starts and the station computes a random value called back off time, in the range of 0 and minimum size of contention window(CW_min_). The minimum contention window value is doubled each time a collision occurs until it reaches the maximum contention window value (CW_max_). A small contention window value decreases the access delay but increases the probability of the collisions.

Since the EDCA mechanism uses *Carrier Sense Multiple Access* (CSMA) which is a probabilistic approach for accessing the medium, hard QoS guarantees, even for the highest priority class, cannot be provided. Among the three main Medium Access Control (MAC) parameters in wireless LAN, TXOP is the most influential, allowing a station to transmit several frames consecutively after winning the channel. In fact, TXOP reduces the MAC overhead since a station can send multiple data frames without contending for the channel between the transmissions.

For each AC, the default values of AIFS, CW_min_, CW_max_ and TXOP limit are fixed in the standard. In other words, the effects of channel variations and the number of opponent nodes have not been considered in the standard and they remain as open research issues. In addition, simulation results indicate that tuning the TXOP limit with respect to the burst size distribution can significantly improve the network performance and the burst delay [3]. Although the allocation of long TXOPs improves the medium utilization and increases the network stability region, some unfairness and security breach problems may arise. It is also shown that under bursty traffic, the TXOP limit plays an important role in avoiding lower priority traffics starvation [3]. Several studies have shown the most effective parameters on TXOP as: network load, mean data rate, maximum burst size, peak data rate, user priorities, minimum physical rate, delay bound, maximum service interval, MSDU size and channel conditions. Therefore, several TXOP adaptation algorithms have been presented [4]–[13]. Most of the works reported in the literature are heuristic without a rigid analysis and consideration of the effect of mutual node interaction. In fact, taking a long TXOP by a node increases other nodes delay and causes them to do the same action which in turn brings about very long delays for all of them. So, each node has to consider the effect of its actions on the others and the overall network performance. Therefore, a distributed TXOP adaption to provide a satisfactory QoS requirement for each queue is still an open issue. As such, game theory based approach, which has not been also investigated in the previous works, seems to be applicable in TXOP adjustment. To achieve this goal, a dynamic TXOP tuning method based on a cooperative game and corresponding to the QoS requirements, the number of frames in each queue and the number of nodes in each AC is proposed in the present study.

The present article is organized as follows: Background of the study including an introduction of the game theory and the related works are provided in section 2. In Section 3, statement of the problem focusing on challenges in the game theory framework as well as game formulation and solution are presented. Numerical results are given in the fourth section. Finally, the article is concluded in section 5.

## 

### Related works

Several techniques have been developed to improve WLAN QoS performance, mainly introducing a trade-off between performance and standard compatibility. A dynamic TXOP adjustment scheme according to the current state of nodes’ transmission queues, in order to adapt to the bursty nature of self-similar traffic types, is presented in [4]. Authors of [5] have designed a distributed TXOP adaptation algorithm to set TXOP limits for given throughput requirements. In [5] each node measures its throughput in a window and compares it with a target value and then tunes its TXOP using the result of this comparison. Another TXOP configuring method, based on varying data rates and collision ratios, which provide the required QoS, is presented in [6]. The method adapts the TXOP limit to the varying PHY rates, channel conditions and network loads. In [7], by taking into account the demands of other nodes; maximal residual unused bandwidth is reclaimed. The authors in [8] introduce a Dynamic TXOP (DTXOP) algorithm which enhances fairness between upstream and downstream resource allocations in Wi-Fi networks. In [9], the dynamic traffic prioritization scheme is proposed for multi-hop wireless networks. The algorithm assigns priorities to each traffic flow based on network status and delay requirements. It also allocates dynamic TXOP based on the precise channel condition prediction. The study of [10] investigates temporal fairness provisioning in multi-rate WLANs and it is shown that equalizing the channel access time causes throughputs to accord with the node transmission rates. In [11], [12] it is shown that there exist some tradeoffs between fairness and system efficiency. Next, a rate adaptive TXOP is proposed to improve fairness. To overcome the degraded performance which resulted in channel errors, in [13], a dynamic TXOP allocation method is proposed. Based on the precise channel condition prediction, the method assigns different TXOPs to different traffic flows.

Finally, in [14], possible misbehaviours in 802.11e including “taking a long TXOP by a malicious node” are investigated and a punishment for such a behaviour is considered to provide fairness among the active nodes. Regarding the above-mentioned studies, while various methods have been used to adjust TXOP, to the best of the authors’ knowledge, the problem of TXOP adjustment has not been investigated in a game theory framework. The following section introduces the proposed method in this study.

## Methods

### Proposed Method

Optimum TXOP allocation mechanism increases channel utilization. Although exploiting longer TXOPs in a network improves channel utilizations and increases the network stable area, it causes low-priority traffic flows which starve and suffer from unfair delays. Therefore, inappropriate adjustment of TXOP brings about unfairness and also some vulnerabilities toward probable maliciousness [3], [15], [16]. In fact, in heavy load traffic, exploiting long TXOPs by some selfish wireless nodes increases the Head of Burst (HoB) delay of other wireless nodes. In other words, choosing a long TXOP by a node has negative impacts on its neighbouring nodes. Imitating the same action by the other nodes leads the network to violate QoS requirements of some applications. So, in adjusting TXOPs, each node has to investigate how its action affects other nodes in the network. Therefore, to consider the impact of nodes on one another, which has been neglected in the previous studies, game theory seems to be an appropriate framework.

Game theory is a mathematical tool that examines decision making in a shared environment with multiple decision makers who have various objectives in mind. Problems of interest involve multiple participants who have individual objectives related to some shared resources. Since game theory arose from the competitive scenario analysis, the corresponding problems are called *games,* the participants of the scenarios are called *players,* the players’ actions are referred to as *moves*, and a sequence of moves is called a *strategy.* Nodes of a network are good examples of such a situation so, game theory is highly applicable to wireless mesh and Ad hoc networks.

Game theory has been widely used in wireless communications [17]–[26]. In CSMA networks, particularly, game theory is exploited for different purposes such as contention control, channel access, power control, and data rate control [27]–[30].

In addition, as game theory is an interesting tool for the analysis of contention-based environments, it is highly applicable in the EDCA mode of IEEE 802.11e in which nodes access the channel through *CSMA* access method. As in CSMA, since accessing a node to the wireless channel influences the access of its neighbouring nodes, game theory can be used for determining the TXOP limits of the nodes in EDCA. The main contribution of this study is to provide a QoS-capable mechanism to determine TXOP using a game theory framework. For this purpose, a non-cooperative game called GTXOP is defined to determine adaptive TXOP. Next, based on the mentioned properties of the game theory a new distributed method to determine TXOP is proposed.

In GTXOP, each wireless station is considered as a game player choosing its TXOP limit as its action. The winner station can transmit several successive frames as long as the transmission time does not exceed the TXOP limit. The proposed game should have an equilibrium state wherein each player should obtain a fair part of game utility. Moreover, the dynamism of the game should be resilient against selfish actions. That is because in autonomous wireless networks it is possible that players choose their actions selfishly to improve their performance regardless of the harm imposed to others. In other words, the proposed game should pursue a converging strategy in a way that no selfish player has the incentive for defying the protocols.

If the number of nodes is small, choosing long TXOPs will help to improve channel utilization. However, as the number of nodes increases, this action will be destructive to all the players in terms of increasing HoB delays. In such a situation, each node has to choose a shorter TXOP in order to decrease the channel access delay. Therefore, TXOP has to be determined dynamically based on the network circumstances.

### GTXOP Formulation

Having an important role in the game mechanism, a payoff function usually consists of utility and cost functions. Since each node is expected to receive a reward for a fair play, the utility can be viewed as a criterion for measuring the satisfaction level of a player. To prevent selfishness, a cost function should be considered for the payoff function as well.

To contemplate a challenging situation, we assume the network to be heavily loaded, meaning that there is always at least one packet awaiting transmission in each node’s queue. Moreover, equi-nodes are assumed such that all the nodes have the same EDCA parameters (i.e. AIFS, CW_min_ and CW_max_) and an ideal physical environment with no error or hidden node effect is considered. For simplicity, the nodes are supposed to hear each other and support only one traffic type with the same mean packet lengths (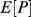), where *P* is the MAC layer payload size and 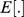 denotes the expectation operation.

Different game strategies based on the network requirements at the MAC layer can be designed by defining different payoff functions. However, the contention between the nodes has to be reflected in the nodal payoff function. From the standpoint of the game theory, the existence and uniqueness of the proposed game is crucial. On the other hand, as a MAC layer protocol, some other aspects should be considered in the utility function as well. For example, the payoff function should converge to an efficient equilibrium point; the fairness problem at the MAC layer should be considered in the payoff function, and a cost function should be included in the payoff function for the efficient use of network resources.

In GTXOP, each node would like to maximize its access to the channel. Being the normalised saturated throughput of node *i*, *S_i_* is defined as the fraction of time the channel is used to successfully transmit payload bits and can be expressed as follows [31]–[36]:
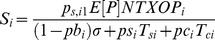
(1)where, *NTXOP_i_* and 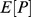 represent the maximum number of frames that node *i* can transmit in each access to the channel and average packet payload size, respectively. So, the mean number of successfully transmitted bits in a transmission period will be 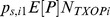, where 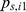 is the probability that a station accesses the channel successfully, and is given by:



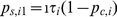
(2)In (2), 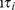 denotes the transmission probability of station *i* and the collision probability due to external collisions is, 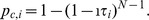 From the network point of view, the probability that a node can successfully access the channel can be written as[31], [33]–[36]:
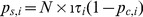
(3)


It is assumed that each station supports only one traffic type, so there is no internal collision. The probability that a station senses the medium busy around itself is defined as 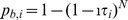 [31], [33]–[36].

**Figure 1:**
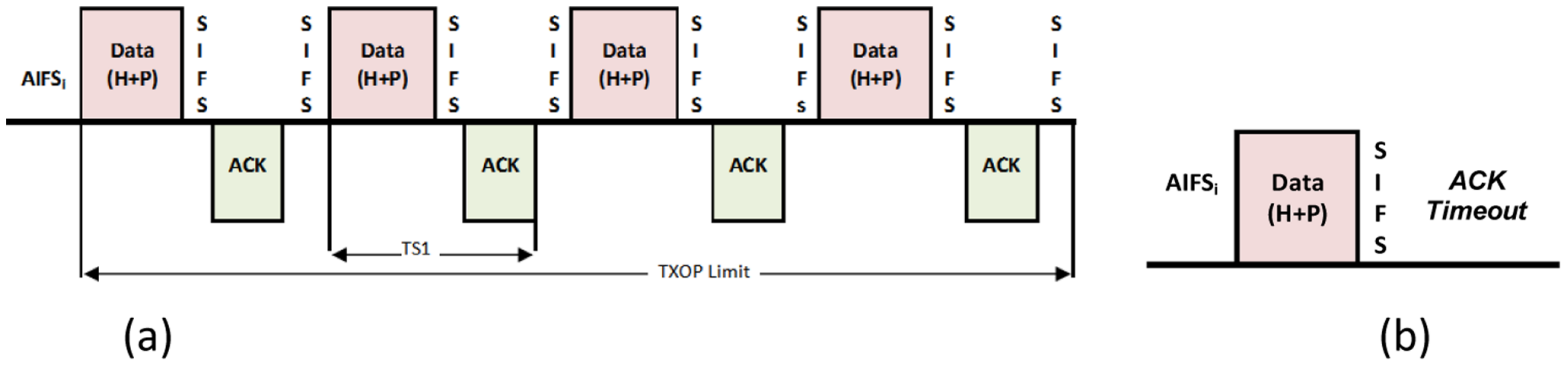
Transmission Time in the basic access mode: (a) Successful transmission time. **(b) Collision time.**



The average length of a transmission period or the channel occupancy time, called logical time slot 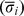, consists of three different components: (1) the channel is idle with probability 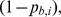 (2) a successful transmission occurs with probability 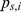 and (3) a collision happens with probability 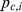. The successful and the collision transmission times depend on the channel access mode (basic or RTS/CTS). In the case of the basic access mode (Figure 1.), 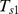 and 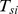 represent successful transmission time of one data frame and a burst transmission time, respectively. A successful transmission burst using the TXOP mechanism consists of multiple-packet transmissions and their ACKs [31], [33], [36]:
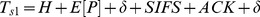
(4)

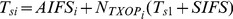
(5)where *H*, *P* and 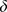 are the times which are spent to transmit the headers of PHY and MAC layers, frame payload and propagation delay, respectively. Note that the PHY header is always transmitted in basic rate, while MAC data frames, including the MAC header and payload are transmitted in data rate [2]. Collision time in basic mode is given as:

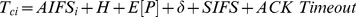
(6)Hence, logical time slot can be defined as:
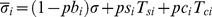
(7)where, 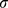 is the time duration of a physical slot. According to (1), 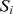 indicates that the higher the 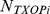, the more the medium utilization. It is worthwhile to investigate the effect of using long TXOP by one player on the other players. It is remarkable that if the nodes use long TXOPs, the channel utilization would be maximized because of reduction in contention time percentage. However, this strategy leads to long HoB delay.

For EDCA which is proposed in order to support QoS, excessive delay may cause significant packet loss for delay sensitive traffic. Per se In GTXOP, the time interval between two successive successful frame transmissions is considered as the cost function.

The time it takes from the moment that an HoB frame reaches the head of a node’s transmission queue to the moment the node wins the contention and the HoB frame is ready to transmit or it is discarded as a result of transmission failures, is called the channel access delay.

Normally an HoB frame after experiencing a number of collisions (*h*) may be successfully transmitted. So, the channel access delay consists of two components: delay from (*h*) collisions and delay from 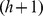 backoff stages. Thus, the average channel access delay, 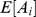, can be obtained as:
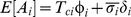
(8)where 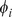 and 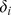 are the average number of collisions before a successful transmission and the average number of time slots that the station defers in backoff stages, respectively and can be written as;
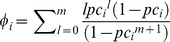
(9)

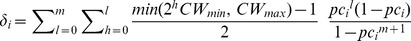
(10)


In addition, some malicious nodes may try to waste the channel by taking an extra-large TXOP and transmitting dummy frames. As such, bounding the TXOP by a proper limit is helpful. The limit should be calculated having the maximum tolerable delay enforced by real-time applications. Based on the above justifications, the payoff function of GTXOP is expressed as:
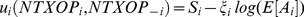
(11)where, 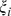 is the weighting factor. In addition to being a normalizing factor, this term provides some trade off between throughput and delay. In (11), 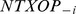 denotes the maximum number of frames that can be transmitted in each access to the channel by other nodes except node *i.*

The first term in the payoff function 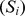 represents that the greater the TXOP, the more the node’s gain from the channel will be. However, by increasing the number of contending nodes (opponents) in the network, the success probability and consequently, the throughput will decrease. The second term indicates to the HoB frame delay which the node is experiencing.

Through maximizing its payoff function, each node can determine its TXOP. It is notable that maximizing this payoff function leads to the maximization of the throughput and also the minimization of the cost related to the HoB delay. The GTXOP is formally defined as a non-cooperative game as follows:

The TXOP game G is defined as below:
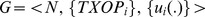
(12)where *N* is the number of active nodes considered as players, 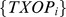 is the set of actions of player *i* and 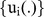 is the set of payoff functions of the players. The objective of GT XOP is to find the TXOP limit of each node at each channel access, during which the winner station can transmit several successive frames as long as the transmission time does not exceed the TXOP limit.

In addition to preventing large TXOPs which result in excessive delays, the strategy space of each node may be constrained to 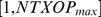.

### Game Solution

In a game, the point where all players make their decisions and an outcome is reached is called as Equilibrium. The most popular equilibrium is Nash in which none of the players gain any benefit via changing their strategies on their own part. To solve a game, i.e. to reach its Nash equilibrium, a number of methods such as Best Response, Gradient and Jacobin can be used.

So, in order to prove the existence of Nash equilibrium, it has to be proved that the payoff function is strongly concave. Assuming 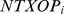 to be a continuous variable, when 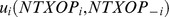 is a twice-differentiable function, if the second derivative 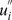 is negative, 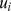 is concave. If new variables *B, C* and *M* are defined as follows:
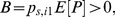
(13)

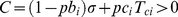
(14)

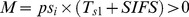
(15)



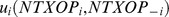
 can be rewritten as:
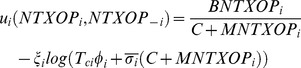
(16)


After twice differentiating 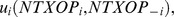 one gets to:
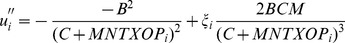
(17)


As 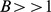 and 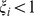, 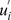 will be negative 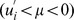 and 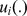 is strongly concave.

Since the payoff function 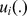 is continuous and strongly concave, there exists the Nash equilibrium [37].

The Nash equilibrium study of a game can be used to estimate the payoff of GTXOP where each of the player’s decisions is based on self-optimization. At the Nash equilibrium, no player can improve its payoff by making changes in his decisions individually. To reach the Nash equilibrium, each node has to maximize its payoff function by solving the following optimization problem:
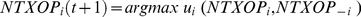
(18)

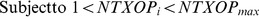
(19)


Assuming 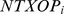 as a continues variable, by differentiating 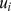 in terms of 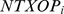, and equating the derivative to zero 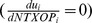, the best 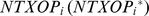 can be calculated.

The simplest method to update strategy mechanism is the best response in which at each stage, every node chooses the best response to the actions of all the other nodes in the previous round. So node *i*, at stage (*t*+1) chooses its TXOP as follows:
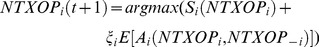
(20)


If these dynamics result in a steady state, then this state is the Nash equilibrium. As the best response convergence is questionable, in most of the games, the Gradient Method which is called *the better response* is exploited [38]. Using Gradient Method, every node tunes its TXOP gradually in a gradient direction based on other players’ actions. Mathematically, this can be written as [38]:
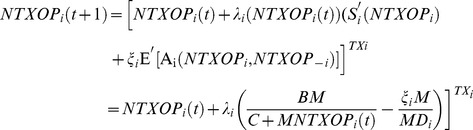
(21)


In (21), 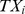 is the strategy space of node *i,* 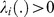 is step size for node *i* which can be a function of node *i* strategy, and *MD_i_* is the media access delay of node *i*. After calculating 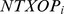, the final TXOP can be obtained as:
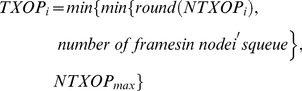
(22)


The Gradient play has an interesting economical interpretation. HoB delay can be considered as the channel access cost, so if the marginal utility of node *i* is greater than its price, the node *i* increases its 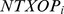 and if the marginal utility is less than the price, the node *i* decreases the 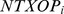. In GTXOP, a node experiencing lower delay has to pay more cost applied by the logarithmic function. A node with short delay has to pay more, proportional to the inverse of its tolerated delay.

The first assumption in game theory, rationality of players, states that all the players follow the rules of the game. Even a malicious node that plays the game correctly but tries to spoil the network by transmitting dummy frames to the channel has to adjust its TXOP to the value, specified by the GTXOP rules. Therefore, it only wastes the opportunity dedicated to it.

This justification is not true for a malicious node which deviates the game rules by taking very large TXOPs to collapse the network [30]. In this case, extra measures should be taken to efficiently detect and penalize that malicious node such as jamming and interrupting its transmission upon grabbing the channel.

The proposed algorithm for GTXOP is given in Table 1. The numerical results of the proposed method and the related discussion are presented in the next section.

**Table 1:** The GTXOP Algorithm.

(1) Initialization: set 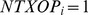 for all the nodes	
(2) Information gathering and game state estimations : do After each transmission	
• Count the total number of successfully transmitted data frames	
• Count the total number of unsuccessfully transmitted data frames	
• Count the total number of experienced time slots	
• Assuming an ideal channel, estimate the collision probability (*pc*), transmission probability 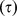 and the number of competing nodes using the following relations [39].	
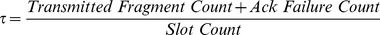	(23)
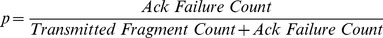	(24)
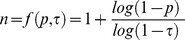	(25)
• Measure maximum delay of frames in its queue (HoB).	
(3) TXOP update: Update TXOP using (21),(22).	

## Results and Discussion

In this section, to validate the proposed method numerical results are presented. For this purpose, different scenarios with different numbers of the nodes are assumed in which each node has one active queue with a finite size. The channel capacity is considered to be 5.5 Mbps and the basic mode is used to access the channel (Figure 1.). The system parameters are shown in Table 2.

**Table 2:** Simulation Parameters.

Mean frame payload	1024B	PHY header	192 bits
**MAC header**	224 bits	ACK	112bits +PHY header
**Channel rate**	5.5 Mbps	Basic rate	1 Mbps
**Physical time slot**	20	CW_min_	63
**Transmission power**	0.05	CW_max_	1023
**PHY Layer**	Direct sequence	SIFS	10 µs

To investigate the accuracy of the proposed method, various numbers of nodes with EDCA parameters, as shown in Table 2., are considered. Next, the results obtained from the proposed method are compared with the TXOP limits of AC3 which is set to 3264 µs. The TXOP setting is evaluated for the nodes transmitting the delay sensitive traffic such as voice in IEEE 802.11(e)(s) WLAN.

**Figure 2:**
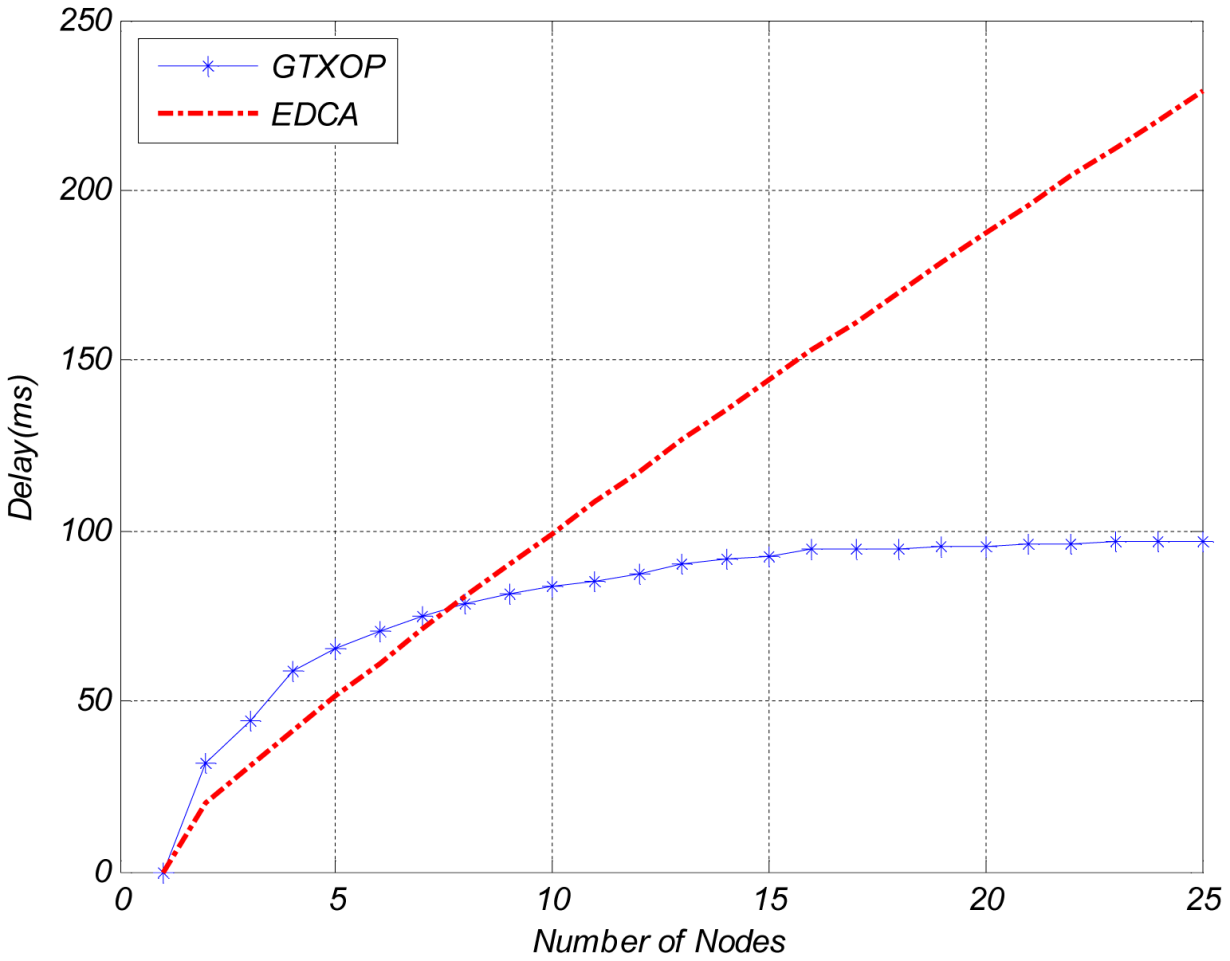
Throughput Comparison of GTXOP and EDCA.

**Figure 3:**
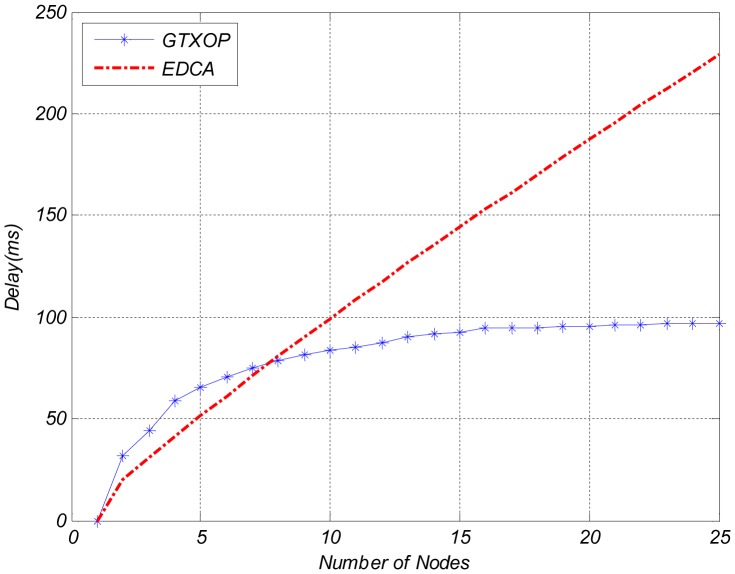
Delay Comparison of GTXOP and EDCA.

Figure 2. compares the EDCA throughput and the throughput of the proposed method per each node. In case of a smaller number of nodes, the nodes can improve their throughputs by choosing longer TXOPs while the delay requirement is provided. But due to the increase in the number of nodes, the network load grows and the nodes have to select shorter TXOPs to provide fair and short HoB delays. So, the nodes throughputs are reduced. Delay variations of the nodes are shown in Figure 3.

As shown in Figure 3., under light load in the network (about less than 8 nodes), delay is small. But with an increase in the number of nodes, delay is enhanced. In this case, the nodes have to use shorter TXOPs to provide delay requirements and also avoid unfairness. The numerical results demonstrate that the delay of the proposed method is lower than EDCA.

When the number of contending nodes is small, contention is also less and the HoB delay is reduced in turn. In fact, the time interval between the two successive transmissions is short. But by increasing the number of nodes, the collision probability increases and consequently, the delay is increased. As it is assumed that all the nodes always have frames to transmit, TXOP adjustment does not have a significant effect on the re-try exceed drop probability. Note that, to reduce collision probability by increasing the number of nodes, CW_min_ has to be tuned in terms of the number of nodes.

Furthermore, if the load increase causes the arrival rate to be close to or greater than the departure rate, the nodes’ queue will saturate immediately. Hence the TXOP adjustment may not be sufficient and an admission control mechanism has to be exploited.

### Conclusions

Adjusting TXOP has significant effects on EDCA performance in wireless LANs. In this paper, using game theory and based on the analytical models of EDCA, a new method for the dynamic adjustment of TXOP values in IEEE 802.11 (e)(s)- EDCA mode was proposed. In GTXOP, each node would like to maximize its access to the channel. A combination of anode’ throughput and time interval between two successive successful frame transmissions of the node is considered as the node’s payoff function.

Numerical results also validated the accuracy and improvement of the proposed method. It is shown that under light load in the network (small number of contending nodes), contention is also less and nodes can choose longer TXOPs to improve their throughput. But with increase in the number of nodes, delay is increased. In this case, the nodes have to use shorter TXOPs to provide delay requirements and also avoid unfairness.
